# General and local predictors of mandibular cortical bone morphology in adult females and males: the seventh survey of the Tromsø Study

**DOI:** 10.1007/s00784-023-05263-0

**Published:** 2023-09-22

**Authors:** Anna Teterina, Sanyalak Niratisairak, Bente Morseth, Napat Bolstad

**Affiliations:** 1 Department of Clinical Dentistry, Faculty of Health Sciences, University of Tromsø–The Arctic University of Norway, Tromsø, Norway; 2 Department of Orthopaedics, Institute of Clinical Medicine, Faculty of Medicine, University of Oslo, Oslo, Norway; 3 Biomechanics Laboratory, Rikshospitalet, Division of Orthopaedic Surgery, Oslo University Hospital, Oslo, Norway; 4 School of Sport Sciences, Faculty of Health Sciences, University of Tromsø–The Arctic University of Norway, Tromsø, Norway

**Keywords:** Mandibular cortical width, Mandibular cortical index, Panoramic radiographs, Osteoporosis, Dual-energy x-ray absorptiometry

## Abstract

**Objectives:**

To analyze factors predicting mandibular cortical width (MCW) and mandibular cortical index (MCI) in adult females and males.

**Material and methods:**

Data on 427 females and 335 males aged 40–84 from The Tromsø study: Tromsø7 were used. *T*-score, age, menopausal status (for females), remaining teeth, and periodontal status were analyzed in linear and logistic regression analyses as predictors of MCW and MCI, respectively.

**Results:**

*T*-score, age, and the number of remaining teeth significantly predicted MCW in females but not males. Standardized *β* coefficients were 0.286, −0.231, and 0.131, respectively. The linear regression model explained 24% of MCW variation in females. MCI in females was significantly predicted by *T*-score, age, and remaining teeth with the Wald values of 9.65, 6.17, and 5.83, respectively. The logistic regression model explained 16.3−23% of the variation in MCI in females. In males, *T*-score was the only significant predictor of the eroded cortex, and the logistic model explained only 4.3–5.8% of the variation in MCI.

**Conclusions:**

The *T*-score demonstrated a stronger relationship with MCW and MCI than other factors in females, which supports the usefulness of those indices for osteoporosis screening. Conversely, the *T*-score exhibited no association with MCW and remained the only significant predictor of MCI in males, yet to a lesser extent than in females.

**Clinical relevance:**

Understanding factors affecting mandibular cortical morphology is essential for further investigations of MCW and MCI usefulness for osteoporosis screening in females and males.

## Introduction

Osteoporosis is a chronic, non-communicable disease that deteriorates bone tissue and makes bones fragile and prone to fractures which are considered a public health problem due to increased mortality risk and considerable health costs [1–3]. Genetic factors and sex define bone mass and structure to a great extent, and osteoporosis is more prevalent in females than in males [3, 4]. Both males and females reach their peak bone mass approximately in their 20s; after that, gradual bone loss starts in both sexes in their third decade due to reduced osteoblast activity [3, 5], and accelerates faster in females in post-menopause due to declining estrogen levels [6].

There are associations between jawbone morphology and the state of bone tissue in the whole body. For example, a moderate positive correlation was found between the mandible’s bone mineral density (BMD), specifically BMD of the buccal mandibular cortex, and femoral neck BMD [7]. Furthermore, the morphology of the mandibular cortex assessed on dental panoramic radiographs was associated with skeletal bone turnover in elderly females [8]. These findings were supported by further research collected in two systematic reviews showing that changes in mandibular cortical morphology on dental panoramic radiographs could predict low BMD or osteoporosis in women [9–11]. Two radiomorphometric indices, i.e., mandibular cortical width (MCW) and mandibular cortical index (MCI), were extensively studied as potentially useful for osteoporosis screening [12].

Nevertheless, little is known about the extent to which different factors are associated with the morphology of the mandibular cortex, while this knowledge is essential for supporting or arguing against using radiomorphometric indices for osteoporosis screening. Such studies are specifically lacking in males [13, 14]. Some studies have shown that age and gender are significant predictors of the thin and eroded cortex; however, their analyses did not account for T-score or other possible factors [15, 16].

Mechanical loading is also important for building and maintaining bone tissue [17]. An animal study showed that rats with a soft diet had lower BMD of mandibles than rats eating solid food [18]. Dental practitioners observe alveolar bone loss after tooth extraction, although it occurs to various extents in different individuals [17]. Tooth loss may affect parts of the mandible other than the alveolar ridge; Taguchi et al. found that the number of remaining teeth adjusted for age was related to mandibular cortex morphology in women [19]. Dutra et al. found an association between remaining teeth and cortical thickness in the antegonial region of the mandible, which might confound relationships between cortical thickness and osteoporosis [20].

One of the most common reasons for tooth loss is periodontitis — an inflammatory disease affecting tooth-supportive structures called periodontium. Dental plaque bacteria induce inflammation in the periodontium, which is subsequently modified by a host immune response. Inflammatory cells release cytokines that activate osteoclasts, while the latter initiate resorption of the alveolar bone surrounding teeth [21]. The mechanistic links between osteoporosis and periodontitis have been studied but remained unclear [22]. There is emerging evidence that patients with periodontitis exhibit a general inflammatory response with elevated levels of C-reactive protein and inflammatory cytokines, including those activating osteoclasts, while osteoclasts are responsible for bone resorption [21, 23–25]. Thus, periodontitis might also be a factor influencing the mandibular cortical bone.

To our knowledge, none of the previous studies have examined the contribution of different factors to the morphology of the mandibular cortex. This study aimed to analyze the relationship between general factors such as skeletal BMD, age, and menopausal status (for females), local factors, such as the number of teeth and periodontal status, and the morphology of mandibular cortex measured by MCW and MCI on dental panoramic radiographs in males and females.

## Material and methods

The Tromsø Study is an ongoing population-based study initiated in 1974 and carried out as repeated cross-sectional surveys in Tromsø, Norway. The data from the seventh survey (Tromsø7) was used in the current study. All inhabitants of Tromsø aged over 40 (*n*=32,591) were invited to participate in Tromsø7, and 21,083 consented, yielding a response rate of 65% [26]. They filled out extensive questionnaires on various health-related topics. All study participants reported their ages and sex, and females reported their menopausal status in the questionnaires. Further information on the Tromsø7 sampling procedure and data collection is available elsewhere [26].

**Fig. 1:**
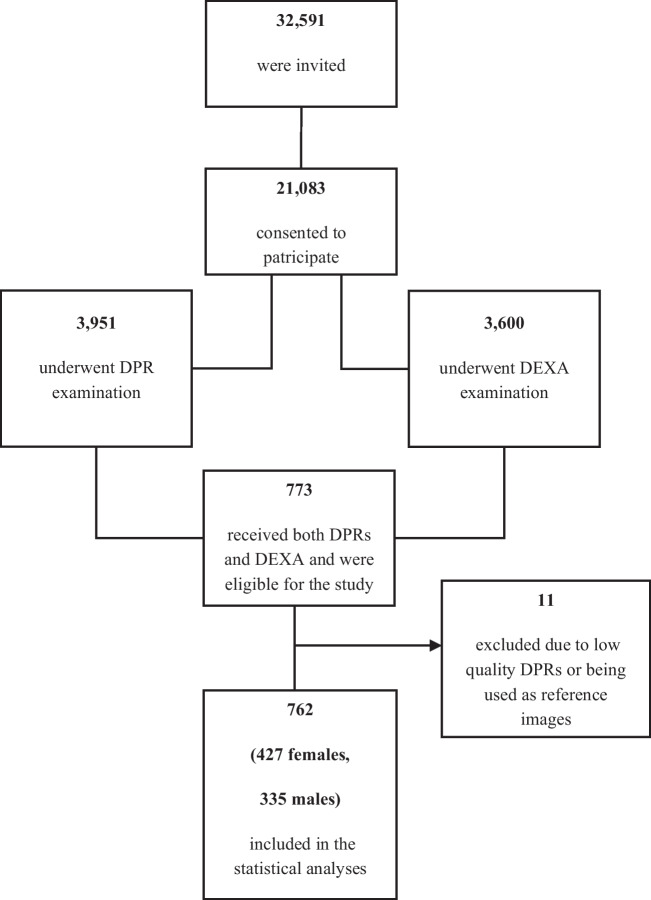
Selection of the study participants

Three thousand nine hundred fifty-one randomly selected participants (Fig. 1) underwent a dental panoramic radiograph (DPR) examination and extensive dental clinical examination during the first visit. Planmeca ProMax 2-Dimensional S3 Dimax-4 panoramic unit (Planmeca Oy, Helsinki, Finland) was used for DPRs acquisition. During a dental examination, bleeding on probing (BOP) and periodontal probing depth (PPD) were recorded at four sites of each toot at all teeth except the third molars. PPD was measured to the closest millimeter with a periodontal probe (WHO probe LM555B). Radiographic marginal bone level (RBL) of interproximal surfaces of all teeth except third molars was measured on DPRs, according to Holde et al. [27]. Periodontitis was defined according to the classification by the American Academy of Periodontology and European Federation of Periodontology, launched in 2017 [28]. Prevalent periodontitis was determined and further classified by stages if RBL was observed at two or more non-adjacent teeth and further classified by stages. Stage I was defined as RBL<15% and PPD ≤4 mm, stage II as RBL of 15–33% and PPD ≤5 mm, or RBL<15% and PPD 5 mm, and stage III–IV as RBL>33%, or <33% RBL and PPD ≥6 mm. Stage III and IV collapsed due to a few cases. Information on the reason for tooth loss, complexity factors, vertical bone loss, furcation involvement, ridge defects, tooth mobility, masticatory dysfunction, and bite collapse/drifting/flaring was unavailable. Non-periodontitis cases were defined as BOP at less than 10% of sites, and gingivitis cases as BOP ≥10% [29]. Periodontal stability was defined as RBL detectable at two or more non-adjacent teeth but no PPD >3 mm.

At the second visit, another 3600 participants, who were randomly selected from the 21,083 Tromsø7 participants attending the first visit, received bone mineral density examination (BMD) using dual-energy x-ray absorptiometry (DEXA) (Fig. 1). Lunar Prodigy device (GE Health-care Lunar, Madison, WI, USA) was used to measure BMD and *T*-scores at the left and right femoral necks. *T*-score is a ratio of the difference between the patient’s BMD and the mean BMD of the young sex-matched adult (reference BMD) to the standard deviation of the reference BMD. This study used a Lunar reference from the US reference population. The *T*-score was expressed in standard deviations (S.D.). The minimum *T*-score from either the left or right femoral neck of each participant was used in this study.

Seven hundred seventy-three participants aged 40 to 84 underwent DEXA and DPR examinations and were included in our cross-sectional study (Fig. 1). Eleven DPRs were excluded due to inferior quality or being used as reference images for MCI assessment. We measured MCW and MCI using ImageJ 1.8.0172 software (U.S. National Institutes of Health, Bethesda, MD, USA) [30] on the rest 762 DPRs. MCW was measured bilaterally along the line drawn through the middle of the mental foramen and perpendicular to the lower mandibular border, as proposed by Ledgerton et al. [31], where MCW is the shortest distance between the upper and the lower borders. In this study, we use an average MCW value of the left and the right side of individuals, and the unit of MCW is a millimeter. Since no reference object was used to control for magnification, all linear measurements were adjusted for a magnification factor of 20%, indicated by the manufacturer. MCI of the cortex was classified into three categories: C1, even and sharp endosteal margin on both sides of the mandible (normal cortex); C2, some endosteal cortical residues, and semilunar defects on one or both sides (mildly eroded cortex); and C3, heavy endosteal cortical residues, the cortical bone is porous on one or both sides (severely eroded cortex). Details of image processing, MCW, and MCI measurements are available from our previous study [32].

Statistical analyses were conducted using IBM SPSS Statistics for Windows version 26.0. MATLAB (version R2021b, The MathWorks Inc., Natick, Massachusetts) was used to make plots. The normality of distribution was assessed by visual examinations of histograms, Q-Q plots, skewness, and kurtosis values. All continuous predictors were normally distributed except the number of remaining teeth. Inter- and intra-observer reliabilities of MCW and MCI were reported in the previous study [32].

Person and Spearman’s correlation coefficients were calculated for normally and non-normally distributed continuous predictors to analyze their correlations with MCW. Hierarchical linear regression analysis was used to analyze relationships among general predictors (T-score, age, and menopausal status), local predictors (remaining teeth and periodontal status), and MCW. Age was added to the first block, *T*-score, and menopausal status were added to the second block, while remaining teeth and periodontal status were added to the third block. Standardized *β* coefficients were used to compare the strength of associations between the predictors and MCW. The linear model assumptions were met, and influential cases were detected in neither females nor males.

Hierarchical logistic regression analysis with the same blocks was used to assess relationships among general, oral predictors, and MCI. MCI was used as a binary outcome (C1 – even and smooth cortex vs. C2, C3 – mildly or severely eroded cortex) due to the low number of participants having the C3 category (55 females and one male). Wald statistics were used to compare the strength of associations between the predictors and MCI. The logistic model assumptions were met.

All regression analyses were carried out separately for males and females. Age was used as a continuous predictor in linear regression and a categorical predictor in logistic regression with the following groups: 40–49; 50–59; 60–69; 70–79; 80+. *T*-score was used as a continuous predictor with a 0.1 SD increment. Periodontal status was divided into three following groups: non-periodontitis or mild periodontitis (health, gingivitis, stage I), moderate periodontitis (stage II), or severe periodontitis (stage III, IV) in both linear and logistic regression analyses. Missing values were excluded pairwise. Data on menopausal status was available for 419 females. Periodontal status for edentulous individuals and individuals with periodontal stability was not included in the regression model, while there were 15 females and 6 males for whom periodontal data was missing. Thus, the total number of females and males having data on periodontal status and included in the regression analysis was 310 and 272, respectively. Other predictors did not have missing values.

## Results

Table 1 presents the characteristics of the study participants by sex. The average MCW was 3.3 mm in females and 4.0 mm in males. Most females (57%) had mildly eroded cortexes, 12.9% had severely eroded, and 30.1% had dense cortexes. Most males (59.1%) had dense cortexes, 40.6% had mildly eroded cortexes, and only one male had severely eroded cortex.

**Table 1:** Characteristics of the study participants

	Females, *n*=427	Males, *n*=335
Mean MCW, mm, (S.D.)	3.3 (± 0.7)	4.0 (±0.6)
MCI		
C1 (dense cortex)	129 (30.1%)	198 (59.1%)
C2 (mildly eroded cortex)	243 (57.0%)	136 (40.6%)
C3 (severely eroded cortex)	55 (12.9%)	1 (0.3%)
Mean age, years, (SD)	66.6 (±8.6)	66.2 (±8.8)
Mean T-score, S.D.s, (SD)	-1.3 (±1.0)	-1.0 (±0.9)
Menopausal status*		
Premenopausal	23 (5.5%)	-
Postmenopausal	396 (94.5%)	-
The median number of remaining teeth	24	24
Edentulous	25 (5.9%)	18 (5.4%)
One or more teeth	327 (76.5%)	258 (77.0%)
Full dentition	75 (17.6%)	59 (17.6%)
Periodontal status**:		
Healthy periodontium, gingivitis or periodontitis, stage I^b^	72 (23.2%)	43 (15.8%)
Periodontitis, stage II^b^	162 (52.3%)	136 (50.0%)
Periodontitis, stage III–IV^b^	76 (24.5%)	(34.2%)

^*^Number of observations=419

^**^Number of observations in females=310; in males=272

**Fig. 2:**
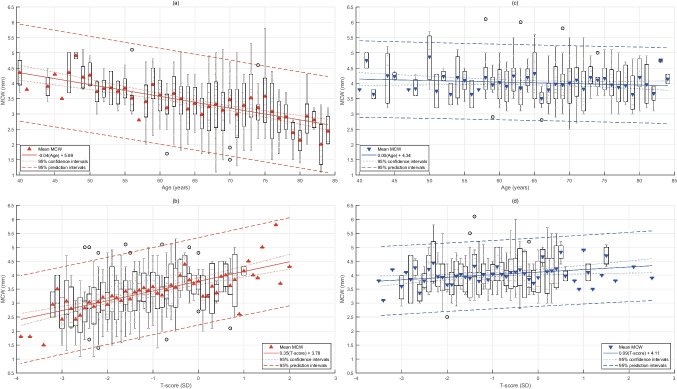
Box plots show mean MCW (filled triangles), its confidence intervals (boxes), and its distribution (vertical lines) in different ages in females (**a**) and males (**c**) and for different T-scores in females (**b**) and males (**d**). Solid lines are regression lines, while dotted lines are confidence intervals for the regression lines

All continuous predictors were significantly correlated with MCW in females (Table 2). Age showed a negative correlation with MCW, with a coefficient of −0.38. Figure 2a presents the means and the distributions of MCW in females by age, showing a tendency to have a thinner cortex with age. *T*-score and the number of remaining teeth showed positive correlations with MCW with coefficients of 0.40 and 0.34, respectively (Table 2). Figure 2b shows a tendency for a thinner cortex with a decreasing *T*-score. In the simple linear regression analyses in females, all predictors except periodontal status were significantly associated with MCW, as suggested by unadjusted *β* coefficients with their confidence intervals (Table 2). In multiple linear regression analysis, *T*-score, age, and the number of teeth remained significantly associated with MCW. Every 0.1 SD lowered *T*-score resulted in 0.25-mm thinner MCW. One year increase in age was associated with a 0.02-mm reduction in MCW in females. Each additional remaining tooth was associated with having a 0.01-mm thicker cortex. *T*-score contributed the most to the variation in MCW in females, with the standardized *β* of 0.286, followed by age and remaining teeth with the standardized *β*’s of −0.231 and 0.131, respectively. The multiple linear regression model for females significantly predicted MCW (*F*=18.7, *p*<0.001). The model explained 24.0% of the variation in MCW in females (*R*^2^=0.240).

**Table 2:** The results of simple (unadjusted *β*) and multiple (adjusted *β*) linear regression analysis predicting MCW in females and males

Predictors	r	Unadjusted *β*, 95% CI	S.E.	Adjusted *β*, 95% CI	S.E.	Standardized *β*	Characteristics of the model
Females							
T-score	0.40**	0.35 (0.27; 0.43) **	0.0	0.25 (0.16; 0.35) **	0.05	0.286 **	Constant =0.47 *F* = 18.7 *p* < 0.001 *R*^2^ = 0.240 *R*^2^ adjusted = 0.227
Age	−0.38**	−0.04 (−0.05; −0.03) **	0.00	−0.02 (−0.04; −0.01) **	0.01	−0.231**	
Menopausal status		−0.70 (−0.10; −0.34) **	0.18	0.11 (−0.34; 0.56)	0.23	0.029	
Remaining teeth	0.34**	0.03 (0.02; 0.04) **	0.00	0.01 (0.001; 0.03) *	0.01	0.131*	
Periodontal status		0.00 (−0.14;14)	0.07	0.03 (−0.09; 0.16)	0.06	0.025	
Males							
*T*-score	0.14*	0.09 (0.02; 0.16) *	0.03	0.08 (0.00; 0.16)	0.04	0.123	Constant = 0.39 *F* = 2.3 *p* = 0.059 *R*^2^ = 0.033 *R*^2^ adjusted = 0.019
Age	−0.07	0.00 (−0.01; 0.00)	0.00	0.00 (−0.01; 0.01)	0.01	0.023	
Remaining teeth	0.10	0.01 (0.00; 0.02)	0.00	0.01 (0.00; 0.02)	0.01	0.116	
Periodontal status		0.05 (−0.12; 0.09)	0.05	−0.03(−0.14; 0.0)	0.06	−0. 036	

^*^Results are significant at 0.05 level

^**^Results are significant at 0.001 level

In males, only *T*-score correlated with MCW (*r*=0.14, *p*=0.009) (Table 2). Males did not tend to have thinner cortexes with older age (Fig. 2c). A significant association between the *T*-score and MCW with an unadjusted *β* coefficient of 0.09 was observed in males in simple linear regression analysis. Figure 2d shows that males tended to have a thinner cortex with decreasing *T*-score. However, this tendency was less pronounced in males than females. In the multiple linear regression analysis, none of the predictors showed associations with MCW in males (Table 2). The overall model did not predict MCW in males (*F*=2.2, *p*=0.059).

In females, all predictors except periodontal status showed significant associations with MCI in the binary logistic regression analysis (Table 3), while multiple logistic regression analysis suggests that *T*-score, age, and the number of remaining teeth were significantly associated with C2 or C3. A 0.1 SD reduction in T-score increased the odds of having C2 or C3 by 38% (OR 0.62, 95% CI, 0.46; 0.84). Being a decade older resulted in 1.65 (95% CI, 1.11; 2.44) times higher odds of having mildly or severely eroded cortex in females. Every remaining tooth was associated with a significant reduction of 7% in the odds of having C2 or C3 (OR=0.93, 95% CI, 0.87; 0.98). *T*-score was the strongest predictor of MCI in females, as demonstrated by the Wald statistic value of 9.65. The overall model significantly predicted mildly or severely eroded cortices (*p* <0.001) and explained 16.3–23.0% of the variation in MCI, as suggested by Cox&Snell and Negelkerke tests.

**Table 3:** The results of binary (unadjusted OR) and multiple (adjusted OR) linear regression analysis predicting MCI in females and males

Predictors	Unadjusted OR, 95% CI	SE	Adjusted OR, 95% CI	SE	Wald statistics	Characteristics of the model
Females						
T-score	0.5 (0.40; 0.64) **	0.12	0.62 (0.46; 0.84) **	0.15	9.65 **	Chi-square = 53.87 *p*<0.001 Cox&Snell *R*^2^ = 0.163 Negelkerke *R*^2^ = 0.230
Age (10 years increment)	2.61 (2.00; 3.42) **	0.14	1.65 (1.11; 2.44) *	0.20	6.17 *	
Menopausal status	18.17 (5.9; 62.4) **	0.63	3.22 (0.76; 13.54)	0.73	2.55	
Remaining teeth	0.90 (0.87; 0.94) **	0.02	0.93 (0.87; 0.98) *	0.03	5.83 *	
Periodontal status	0.88 (0.62; 1.26)	0.17	0.76 (0.51; 1.13)	0.20	1.78	
Males						
*T*-score	0.71 (0.56; 0.90) **	0.12	0.73 (0.56; 0.96) *	0.14	5.16 *	Chi-square = 12.01 *P* = 0.017 Cox&Snell *R*^2^ = 0.043 Negelkerke *R*^2^ = 0.058
Age (10 years increment)	1.36 (1.06; 1.76) *	0.13	1.20 (0.8; 1.65)	0.16	1.26	
Remaining teeth	0.95 (0.92; 0.97) **	0.01	0.97 (0.93; 1.02)	0.02	1.32	
Periodontal status	1.09 (0.77; 1.56)	0.18	0.98 (0.67; 1.43)	0.93	0.00	

^*^Results are significant at 0.05 level

^**^Results are significant at 0.001 level

Binary logistic regression analyses in males show a similar result to females in which all predictors except periodontal status were significantly associated with MCI (Table 3). Multiple logistic regression analysis suggests that *T*-score remained significantly associated with MCI with an odds ratio of 0.73 (95% CI, 0.56; 0.96). The overall model significantly predicted mildly (C2) or severely (C3) eroded cortex in males (*p*=0.017) but explained only 4.3-5.8% of the variation in MCI.

## Discussion

This study analyzed the relationship among general (sex, age, *T*-score, menopausal status), local factors (remaining teeth and periodontal status), and morphology of the mandibular cortex assessed by MCW or MCI. All factors together explained the variation in MCW and MCI in females more than in males.

*T*-score was the strongest predictor of MCW and MCI in females, while in males, T-score was the only significant factor associated with MCI. Previous studies mainly assessed the diagnostic efficacy of radiomorphometric indices for osteoporosis screening [9, 11]. Fewer studies explored the association between MCW, MCI, and BMD in females or males [13, 33, 34]. Analyzing the relationships between radiomorphometric indices and BMD and considering relevant confounding factors is necessary to support using these indices for osteoporosis screening, specifically in males, in whom such studies are lacking. Even though we cannot directly compare the odds ratios and *β* coefficients from other studies with ours due to different methods and regression models used, it is evident from previous research that females with lower skeletal BMD have thinner and more eroded cortexes [34–37]. A positive correlation between *T*-score and MCW in females ranging from 0.33 to 0.45 in previous studies was consistent with our findings [34–36]. Our study found no significant association between *T*-score adjusted for other factors and MCW in males. In contrast, two previous studies found an association of thin MCW in males with osteoporosis (*T*-score ≤ −2.5 SD) [13, 38]. Unlike our study, Leite et al. also found a correlation coefficient of 0.29 for MCW and BMD at the femoral neck in males [13]. Nevertheless, MCI was associated with *T*-score in males in this study, which was consistent with Leite et al. [13], while another study did not find such an association [38]. This divergence in findings might be related to smaller study samples, differences in sampling procedures, or a lack of adjustment for other factors in previous studies.

Age was another major contributor to MCW and MCI in females but not males. Our results align with previous studies that found an interaction between age and sex in the way that cortical thickness reduced more prominently in females than males [15, 19, 20, 39]. Similar trends were observed in other studies exploring the bone geometry and cortical thickness at different skeletal sites in relation to age and sex [40, 41]. A plausible biological explanation for maintaining mandibular cortical thickness in older men could be sex hormones, which play a crucial role in bone formation and resorption. Estrogens increase endosteal and reduce periosteal bone formation in females during puberty, while androgens accelerate periosteal bone formation in growing males [4, 5]. These physiological mechanisms contribute to sex dimorphism in the adult skeleton. After a certain age, bone resorption exceeds bone formation at the inner bone surface in both sexes. However, bone formation continues at the outer bone surface faster in males than in females due to androgens. Thus, males maintain their cortical bone not because they lose less endosteal bone than women but due to a greater periosteal formation [5]. Our study also found that age was a significant predictor for MCI in females but not males (Tables 2 and 3). On the contrary, several studies found that MCI became more eroded with age, regardless of gender [39, 42, 43]. At the same time, those studies did not consider other confounding factors, which may partly explain the disagreement between ours and previous findings. However, one of the studies was longitudinal, which strengthened their results compared to ours [43].

In our study, the number of remaining teeth was significantly associated with MCW and MCI in females but not males (Tables 2 and 3). It is well established in previous research that mechanical strains and subsequent osteocyte response define the geometry and morphology of skeletal bones [44–46]. Thus, it would be appropriate to hypothesize that the lack of loading forces in edentulous people or those with fewer teeth can independently influence the mandibular cortex. Several studies have found an association between remaining teeth and mandibular cortical morphology, even when controlling for age [16, 19, 20, 42]. Okabe et al. found that the number of remaining teeth was weakly correlated with MCW equally for both sexes (0.19 male and 0.14 female) [14]. Dutra et al. found that the number of remaining teeth was related to the thickness of the mandibular cortex, irrespective of gender [20]. Unlike Okabe et al. and Dutra et al., we did not find relationships between remaining teeth and radiomorphometric indices in males. Our results were consistent with Taguchi et al., who also found no relationships between remaining teeth on both the upper and lower jaws and MCW in males [19]. However, considering only mandibular teeth might be more logical, like some previous studies, since we assess the mandibular cortex [16, 20]. Despite the significant association in females, remaining teeth contributed to the thin and eroded cortex to a minor extent. Similar to our study, Legerton et al. and Gulsahi et al. showed that the influence of dentition on cortical erosion and cortical thickness in the antegonial region was weaker than that of age [16, 42].

In our study, menopausal status was significantly associated with MCW and MCI in univariate analysis but not after adjustments, meaning that the other factors confounded this association greatly. Unlike our results, two previous studies found an association between menopausal status and MCW and MCI but did not consider other relevant confounding factors [47, 48].

The association between periodontitis and mandibular cortical morphology is poorly studied. Recent systematic reviews have shown that many studies have explored the effect of osteoporosis on periodontal health [22, 49]. However, some researchers hypothesized the opposite relationship. Two longitudinal studies explored the independent effect of periodontal disease on skeletal bone tissue and found an increased risk of osteoporosis among people diagnosed with periodontitis after accounting for confounders [50, 51]. The rationale behind those hypotheses was that patients with periodontitis exhibit higher systemic levels of inflammatory mediators such as interleukin (IL-2, IL-6) and tumor necrosis factor (TNF-α) [24]. Those inflammatory mediators affect the remodeling of bone tissue and may thus increase the risk of osteoporosis development [52, 53]. One can also speculate that periodontitis may influence the mandibular cortex via systemic inflammation mechanisms. Tooth loss and mobility due to severe periodontitis leading to a lack of mechanical loading may also influence mandibular cortex morphology.

Unlike few existing studies on the subject, this study found no relationship between mandibular cortical morphology (MCW and MCI) and periodontal status for females or males [47, 48]. That might be due to the difference in study design, the wide variation of periodontal measurements, and case definitions in the literature, which makes comparisons between studies difficult. A recent study exploring the utility of the new periodontal disease classification found that different classification systems affect association estimates in epidemiological studies to a great extent, and the utility of the new classification is not well-studied [54]. The 2017 World Workshop on the Classification of Periodontal and Peri-Implant Diseases and Conditions came to a consensus that staging in periodontitis diagnosis should be based on clinical attachment loss (CAL), RBL, PPD, and other factors like teeth missing due to periodontitis [28]. First, CAL and the reason for tooth loss were unavailable in our study. Had we obtained this additional information, the classification of study participants into the periodontal disease categories would probably have changed. Second, PPD is a parameter that reflects the extent of current inflammatory processes, while RBL reflects patients’ periodontal disease experience in the past. It is unclear which parameter is most appropriate when exploring the link between periodontal disease and mandibular cortical morphology. Common sense suggests that cortical bone loss does not occur quickly under an inflammatory process; therefore, RBL as a sign of periodontitis history rather than PPD should be used to explore the abovementioned link. In support of this statement, the systematic review of the association between periodontitis and osteoporosis found that most studies using radiological criteria to define a periodontal case showed significant associations with osteoporosis. At the same time, results were more controversial for studies using clinical measurements for case definition [22].

Another factor that can potentially influence mandibular cortical morphology but has not been included in this study is the mechanical bone load produced by masticatory muscles. It has been previously reported that masticatory load affects both the trabecular and cortical bone in different mandibular regions but predominantly in the angle of the mandible [55–57]. Different types of face anatomy are associated with various masticatory loads: individuals with a short face type (hypo-divergent) have a small mandibular angle and short masticatory muscles with increased masticatory function. In contrast, those with long face types (hyper-divergent) have a large mandibular angle, longer muscles, and decreased masticatory function [58]. A study by Gonca et al., published in 2023, found that individuals with hyper-divergent type exhibited less dense trabecular bone at ramus, condyle, sigmoid notch, and mandibular angle, while mandibular cortical width was thinner only in the second molar projection [59]. Since only DPRs were available in this study, while lateral cephalometric radiographs are commonly used to assess vertical facial type, we could not assess the potential association of masticatory load with the morphology of the mandibular cortex. It might be worthwhile to study this association in future research.

The current study has several limitations. First, it is a cross-sectional study. Despite the arguments regarding the possible links between sex, bone mineral density, age, remaining teeth, periodontitis, and mandibular cortex, we cannot infer the directions of these relationships. Another limitation is the suboptimal inter- and inter-observer agreement of radiomorphometric indices reported in our previous work [32]. Moreover, the panoramic radiographs in this study were not standardized using reference objects, i.e., we could not make precise corrections of MCW for magnification. In addition, the classification of periodontitis is likely to be biased because RBL measurements were performed on DPRs, which distort spatial relationships between anatomical structures to some extent. At the same time, the superimposed cervical spine often hinders bone measurements in the anterior region of the jaws. Suboptimal observer agreement, spatial distortion inherent to DPRs, and image distortion due to the patient’s head misalignment were likely to produce random errors and a substantial unexplained variation in mandibular cortical morphology.

## Conclusion

T-score followed by age contributed most to variation in MCW and MCI in females, supporting the idea of using MCW and MCI for osteoporosis screening for females. Nonetheless, neither general nor local predictors explained the variation in MCW in males. Only the *T*-score was associated with male MCI, though the association was weaker than in females. 

## Data Availability

The data supporting this study's findings is available from the Tromsø Study but is not publicly accessible due to licensing restrictions. Researchers affiliated with the institutions with research expertise can access the data upon application to the Data and Publication Committee for the Tromsø Study (https://uit.no/research/tromsostudy).
